# Epidemiology of *Campylobacter* infections among children of 0–24 months of age in South Africa

**DOI:** 10.1186/s13690-022-00850-1

**Published:** 2022-04-02

**Authors:** Amidou Samie, Resoketswe Charlotte Moropeng, Nicoline Fri Tanih, Rebecca Dillingham, Richard Guerrant, Pascal Obong Bessong

**Affiliations:** 1 Department of Microbiology, University of Venda, Thohoyandou, South Africa; 2 Department of Earth, Water and Science, Tshwane University of Technology, Pretoria, South Africa; 3 Department of Medical Laboratory Sciences, University of Buea, P.O Box 63, Buea, South West Region Cameroon; 4 Center for Global Health, University of Virginia, Charlottesville, USA; 5 HIV/AIDS & Global Health Research Programme, University of Venda, Thohoyandou, South Africa

**Keywords:** *Campylobacter spp.*, Prevalence, ELISA, PCR, Young Children, South Africa

## Abstract

**Background:**

*Campylobacter spp.* are one of the most frequent causes of diarrhoeal disease in humans throughout the world. This study aimed at determining the prevalence and the genotypic distribution of *Campylobacter spp.* and their association with diarrhoea and child growth in children of less than the age of two in the Limpopo Province of South Africa*.*

**Methods:**

A total of 4280 diarrheal and non-diarrheal stool samples were collected on a monthly basis from children recruited at birth and followed up to 24 months. All stool samples were screened for the presence *Campylobacter* antigen using ELISA technique after which CAH 16S primer was used on the positive samples to confirm the presence of *Campylobacter*. Subsequently, the PCR positive samples were further characterised using species specific primers for *Campylobacter jejuni* and *Campylobacter coli.*

**Results:**

*Campylobacter* antigen was detected in 564/4280 (13.2%). *Campylobacter* was more commonly found in diarrheal stools (20.4%) compared to non-diarrheal stools (12.4%) with a statistically significant difference (χ^2^ = 7.345; *p* = 0.006). Throughout the year there were two main peaks of *Campylobacter* infection one in December- January and the second peak in June. The prevalence of *Campylobacter* increased with the age of the children up to 11 months after which the prevalence decreased. Out of 564 positive ELISA samples, 257 (45.6%) were confirmed to have 16S rRNA gene for *Campylobacter spp*. Furthermore, *C. jejuni* was found to be more prevalent (232/257) than *C. coli* (25/257) with a prevalence of 90.3% and 9.7%, respectively*.* Both *C. jejuni* and *C. coli* were significantly associated with diarrhea with statistical values of (χ^2^ = 22.224; *p* < 0.001) and (χ^2^ = 81.682; *p* < 0.001) respectively. Sequences generated from the analysis of *hip* gene confirmed the PCR positives samples were *C. jejuni* positive.

**Conclusions:**

This study has delineated a high prevalence of *Campylobacter spp*. in the study cohort. Moreover, *C. jejuni* was found to be more prevalent than *C. coli* both of which were associated with diarrhea. These findings are of clinical and epidemiological significance.

## Background

The importance of *Campylobacter* as a cause of bacterial enteritis has grown tremendously for the past three decades. Moreover, it has been indicated that its prevalence is higher in developing nations than in developed nations even though studies focusing on detection of *Campylobacter spp.* have largely been performed in the developed world. In addition, Coker and co-authors [1] have highlighted that *Campylobacter* infection is mostly implicated as a cause of diarrhoea in the first six months of life in developing country settings with illness/infection ratios decreasing with age [2]. Nonetheless, there are few studies in Africa, which have reported on the prevalence of campylobacteriosis among young children. A study conducted by Randremanana and co-authors [3] in Moramanga, Madagascar reported 8.9% of *Campylobacter* isolation from diarrheic samples while that in non-diarrheic samples was 9.4%.It was estimated that approximately 90% of *C. jejuni* is responsible for human *Campylobacter enteritis* cases and *C. coli responsible* for most of the remaining cases [4].

One of the studies in Tanzania reported the presence of *C. jejuni* in 34.8% of children with gastroenteritis [5]. In addition, another study in Ile-Ife, Nigeria, found that *C. jejuni* is an important agent of diarrhoea in children of less than the age of five [6]. Another study in Cape Town, South Africa reported a prevalence of 40% of *C. jejuni* from children with diarrheal [7]. In Venda, stool samples were examined for the presence of *Campylobacter species* in adults and children with diarrheal, and the prevalence estimates of *C. jejuni, C. coli* and *C. concisus* were 6.2%, 3.1% and 2.8% respectively; however only 39 of the 255 samples analyzed were from children < 5 years of age [8]. Also, in Venda, South Africa, *Campylobacter spp*. was also isolated from 20% of stool samples tested from HIV-positive individuals [9].

Nonetheless, there is still limited information on the prevalence of *Campylobacter spp.* in healthy cohort children in a rural community of South Africa. Moreover, there still a dire need in developing countries to determine whether malnutrition is a risk factor of harboring *Campylobacter* spp. as most of the developing countries are underprivileged with no access to improved safe drinking water. This study therefore aimed at determining the prevalence of *Campylobacter spp* from stool samples of children in South Africa who were part of the MAL-ED study cohort using ELISA coupled with molecular technique and, to determine the prevalence of *C. jejuni* and *C. coli* and their association with diarrheal and child growth. The MAL-ED study has been previously described [10]. MAL-ED was a birth cohort study to investigate the interactions between enteric infections, gut function, physical growth, cognitive development, and vaccine response.

## Methods

### Ethical consideration

The study protocol was approved by the Safety, Health and Research Ethics Committee of the University of Venda, South Africa, and the Institutional Review Board of the University of Virginia, USA. The purpose of the study was explained to the mothers and/or their legal guardian and written informed consent was obtained before sample collection. Only participants who provided signed informed consent were enrolled for the study.

### Study site and sample collection

The MAL-ED South Africa site has been described [11]. Briefly, the study community was rural and of low socio-economic status. Study participants were enrolled at birth or latest 17 days after birth. Children with birth weight less than 3.5 kg, or diagnosed with a congenital disease, or hospitalized for up to two weeks after birth were excluded from enrolment. Enrolment for the study began in November 2009 in the Dzimauli community of the Limpopo Province of South Africa. For the first 12 months of life, stool samples were collected monthly and thereafter every three months until 24 months of age. Additionally, a stool sample was collected whenever there was diarrhea. Diarrhea was defined as the passing of three loss stools in a 24-h period; and diarrhea episodes had to be separated by at least three days of no loss stools. We collected a total of 4280 diarrheal and non-diarrheal stool samples. Of the 4280 stool samples [2145 were from males and 2135 were from females]. Sample collection and transportation was done according to a standard common protocol for the MAL-ED Network [12]. Briefly, after production, stool samples were immediately placed in a sterile wide screwed cap container containing Cary-Blair medium, and transported in a cooler box with ice packs for subsequent laboratory processing within 4 h of collection. Demographic data such as, age and sex of each child were also collected.

### Detection of *Campylobacter* species in stool samples by ELISA

All 4280 stool samples were screened for the presence of *Campylobacter spp* using a commercial enzyme-linked Immunosorbant assay (ELISA) kit, manufactured by ProSpecT (Oxoid Ltd, Wade Road, Basingstoke Hants, RG24 8PW UK),following the manufacturer’s instructions. Briefly, 200μL *Campylobacte*r sample diluent was transferred into the 2 mL microcentrifuge tube (Thermo Fisher Scientific, Johannesburg, SA) containing 0.2 g of stool sample and vortexed for 15 s and 200μL of the diluted stool sample was transferred to the plastic microtiter wells coated with specific monoclonal antibodies. After 60 min of incubation at room temperature, the microwellplate was washed with washing buffer, 3 times and 4 drops (200μL) of enzyme conjugate was added to each microwell and incubated for 30 min at room temperature. The microwell plate was then washed 5 times before adding 2 drops (100μL) of substrate and incubated for 10 min at room temperature. One drop of stop solution was added to stop the reaction and the absorbance was read at 450–630 nm. All samples with an OD suspension greater than 0.170 were considered positive and those that are less than 0.170 were considered negative.

### Molecular characterization of *Campylobacter jejuni and Campylobacter coli*

#### Extraction of Genomic DNA from stool samples

Genomic DNA was purified from 564/4280 stoolsamples which were positive for ELISA using protocol described by Mukherjee et al. [13] with some slight modification. Briefly, 0.2 g of stool sample, frozen at -70 °C, was diluted in 0.5 mL of lysis buffer (Tris–HCL, 0.5 M; EDTA, 20 mM; NaCl, 10 mM; SDS, %0.1; pH 9.0) in 2 mL microcentrifuge tube and vortexed for 15 s. After vortexing, samples were incubated at 65 °C for 2 h; 600µL of phenol–chloroform-isoamyl alcohol (25–24-1) was added to each sample and mixed thoroughly. Samples were centrifuged at 13,400 g for 15 min; the top layer was transferred in to a new 2 mL microcentrifuge tube and 600µL of chloroform was added and mixed well. The samples were centrifuged at the same speed and time, and the supernatant was collected into a new 2 mL microcentrifuge tube. To the supernatant, 50µL of 5 M NaCl and two volume of absolute ethanol was added and kept frozen overnight to allow DNA to precipitate. On the following day, the samples were centrifuged at 13,400 g for 15 min. To the pellet; 600µL of 70% ethanol was added, mixed and centrifuged once more at the same speed and time. The pellet was blot dried on a clean paper towel and 200µL of TE buffer (1 M Tris–HCl pH 8; 0.5 M EDTA) was added and incubated at 45 °C for 3 h. After incubation, the DNA was store at -20 °C for further analysis.

#### Detection of *Campylobacter jejuni and Campylobacter coli* by PCR

Prior to characterization to species level, CAH 16S primer (F5’-AATACATGCAAGTCGAACGA-3’ and R5’-TTAACCCAACATCTCACGAC-3’) was used to confirm the ELISA *Campylobacter* antigen positive samples. Subsequently, *C. jejuni and C. coli* were amplified from PCR confirmed samples using species specific primers as listed in (Table 1). Briefly, the reaction mixture consisted of 5.0μL of DNA, 25μL Dream Taq Green PCR master mix (Inqaba Biotech, Pretoria, South Africa), 0.1 μM primer and nuclease free water was added to make a final volume of 50μL. The PCR conditions were as follows; heat denaturation at 95 °C for 2 min, followed by 35 cycles at 95 °C for 30 s, annealing at 52 °C for 30 s and extension at 72 °C for 90 s, with final extension at 72 °C for 10 min. The PCR products were separated by electrophoresis in 1.8% agarose gel (w/v) containing 8µL of ethidium bromide at 100 V for 45 min in 1X Tris–acetate buffer, visualized by UV-transilluminator.

**Table 1:** PCR primers used to detect *C. jejuni and C. coli* in stool samples

Primer name	Organism detected	Gene name and size (bp)	Sequence (5’-3’) and annealing temperature	References
*Hip* 1a *Hip* 2b	*C. jejuni*	Hippuricase gene, 176	-ATG ATG GCT TCT TCG GAT AG- -GCT CCT ATG CTT ACA ACT GC- 58 °C	Linton et al*.* [14]
CC18F CC519R	*C. coli*	Aspartokinase gene and the downstream ORF of *C. coli*, 500	-GGT ATG ATT TCT ACA AAG CGA G- -ATA AAA GAC TAT CGT CGC GTG- 66 °C	Linton et al*.* [14]

### Gene sequencing analysis

The PCR products of the hippuricase gene used for identification of *C. jejuni* were sequenced (Inqaba, Pretoria, South Africa). Sequences were assembled using Staden package. Sequences were edited using Bioedit package and aligned using Clustal W multiple aligner. A similarity plot was then done to determine points of mutation in line with the reference strain.

### Statistical analysis

The results were analyzed using Statistical Package for the Social Sciences (SPSS version 22.0). The Chi square test was used to compare *Campylobacter* infection from children’s diarrheal and non-diarrheal stool samples, other characteristics of the stool and demographic information like gender. Differences were considered significant at *P* ≤ 0.05. Moreover, in order to indicate a child’s nutritional status, anthropometric indices (height-for-age, weight-for-height, and weight-for-age) were expressed as z-scores. Anthropometric data from reference populations published by the NCHS and United States Centers for Disease Control was used as standards. Anthropometric measurements were converted to z-scores using the EPINUT component of the EPI-INFO computer program version 6.

## Results

### Characteristics of study samples and *Campylobacter* infection

A total of 4280 samples were collected from 290 children over a period of 24 months. Of these samples, 2135 (49.9%) were from females, and 4084 samples were non-diarrheal (Table 2).

**Table 2:** Characteristics of samples collected from the Dzimauli community of the Limpopo Province of South from 2009 to 2012

Characteristics		Frequency	Percent (%)
Gender	Male	2145	50.1
	Female	2135	49.9
Diarrhea	Non Diarrhea samples	4084	95.4
	Diarrhea samples	196	4.6
Mucus	Without mucus	3605	87.42
	With mucus	519	12.58
Blood	Without blood	4123	99.975
	With blood	1	0.02
*Campylobacter* infection	Negative	3716	86.8
	Positive	564	13.2

### Frequency of *Campylobacter infection* using ELISA method

*Campylobacter* infection was frequent in (564/4280) 13.2% of the stool samples evaluated in the present study. Frequency was higher in females 296/2135(13.9%) than in males 268/2145 (12.5%). Diarrheal samples that were found to be positive for *Campylobacter* were 40/196(20.4%) while 85(16.4%) stool samples with mucus were found to be positive for *Campylobacter* (Table 3).

**Table 3:** Characteristics of the samples and *Campylobacter* infection from the Dzimauli community of the Limpopo Province of South from 2009 to 2012

Sample characteristics		*Campylobacter* positive	Percentage (%)	Total
Gender	Male	268	12.5	2145
	Female	296	13.9	2135
Sample type	Non diarrheal	509	12.4	4084
	Diarrheal	40	20.4	196
Sample consistency	Liquid	24	17.3	139
	Watery	4	22.2	18
	Soft	297	13.6	2182
	Formed	239	13.4	1785
Mucus	Non mucus	479	12.7	3761
	Mucus	85	16.4	519
Total positive *Campylobacter* samples by ELISA		564	13.2	4280

### Distribution of* Campylobacter species* by age, season and years

**Fig. 1:**
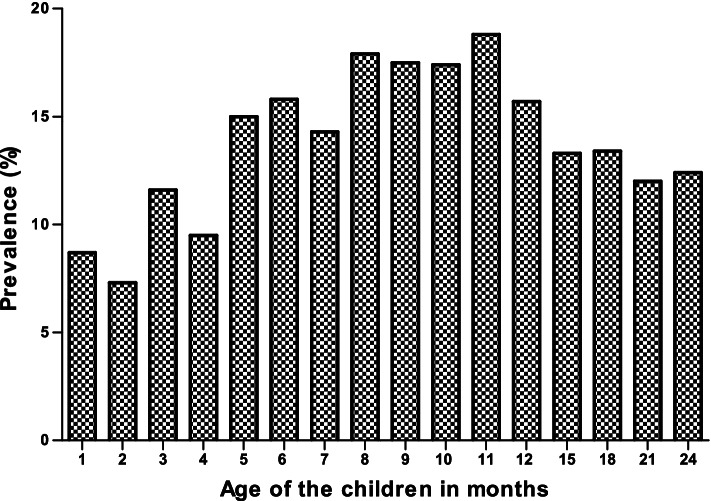
Overall distribution of *Campylobacter* *spp*. by age in the study population

**Fig. 2:**
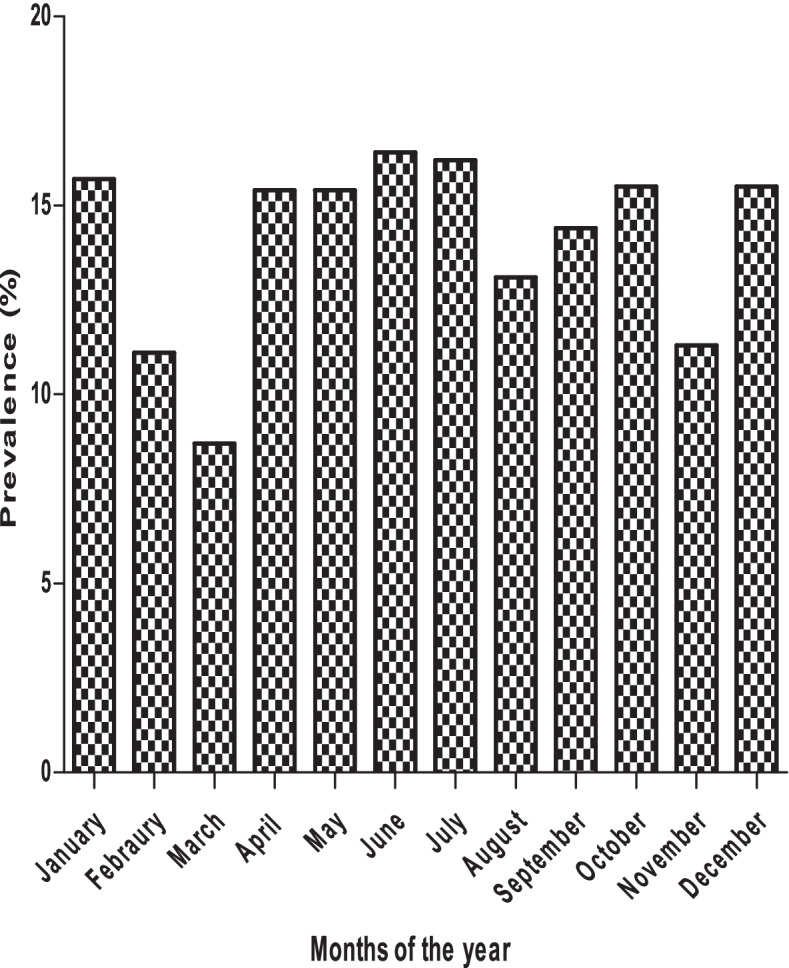
Seasonal distribution of *Campylobacter* *spp*. in the study population

**Fig. 3:**
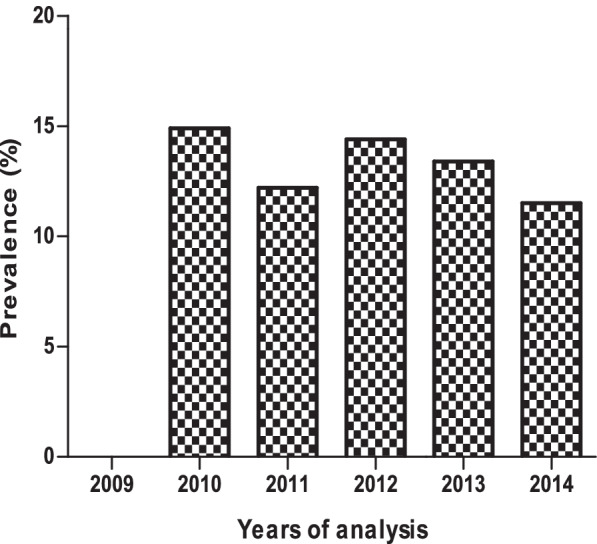
Yearly distribution of *Campylobacter* in the study

The samples were collected from month 1 to month 24. The results show that the *Campylobacter* infection increases as the child grows, with a high prevalence at month 9 and 11. The infection decreases from month 12 and start to increase again at 24 month (Fig. 1). Also, the results obtained showed that *Campylobacter* infection was more common in rainy months (58.1%) than in dry months (47.3%), autumn (31.5%) and spring (27.5%) (Fig. 2). The prevalence of *Campylobacter* infection was high in 2010 (14.7%) compared to 2011 (12.0%). In 2012, *Campylobacte*r infection increased (13.9%) and decreased in 2013 (12.8%). This result confirms that *Campylobacter* infection is endemic in the study area as the prevalence remains almost at the same level (Fig. 3).

### Prevalence of *Campylobacter jejuni and Campylobacter coli* and their distribution and child growth

All ELISA *Campylobacter* antigen positive samples, were confirmed by conventional PCR whereby 16S rRNA gene was detected in 257/564 (45.6%) leaving 307/564 (54.4%) to be negative for the PCR gene probe that is specific to only *C. jejuni* or *coli*, but not other *Campylobacter* strains, that may also be important [15]. For the detection of *C. jejuni* and *C. coli* from human stool samples, species specific primers were used and it was found that *C. jejuni* was more prevalent than *C. coli* with a prevalence of 90.3%(232/257) and 9.7% (25/257) respectively*.* Both *C. jejuni* and *C. coli* infection were more observed in children that had diarrhea than those without with a statistical significant difference of (χ^2^ = 22.224; *p* = 0.000) and (χ^2^ = 81.682; *p* = 0.000) respectively. *C. jejuni* was more common in females (15.2%) than in males (11.8%) and the difference was statistically significant (χ^2^ = 4.190; *p* = 0.041). For *C. coli*, the infection rate was similar between female (1.4%) and male (1.4%) children, and the difference was not statistically significant (χ^2^ = 0.000; *p* = 0.995) **(**Table 4).

**Table 4:** Overall distribution of *C. jejuni* and *C. coli* with child growth from the Dzimauli community of the Limpopo Province of South from 2009 to 2012

**Anthropometric characteristics**		*C. jejuni* Positive	Total	χ^2^	*P*-Value	***C. coli* Positive**	Total	χ^2^	*P*-Value
**Gender**	Male	106 (11.8%)	898	4.190	**0.041**	13 (1.4%)	898	0.000	0.995
	Female	126 (15.1%)	832			12 (1.4%)	832		
**stunted**	Not stunted	164 (13.3%)	1235	0.061	0.801	15 (1.2%)	1235	1.610	0.204
	Stunted	68 (13.7%)	495			10 (2.0%)	495		
**Underweight**	Not underweight	210 (13.2%)	1585	0.423	0.515	23 (1.5%)	1585	0.005	0.945
	Underweight	22 (15.2%)	145			2 (1.4%)	145		
**Wasted**	Not wasted	225 (13.6%)	1655	1.122	0.259	25(1.5%)	1655	1.150	0.284
	Wasted	7 (9.3%)	75			0 (0.0%)	75		
**Diarrhea**	No diarrhea	209 (12.6%)	1658	22.224	**0.000**	15 (0.9%)	1658	81.682	**0.000**
	Diarrhea	23 (31.9%)	72			10 (13.9)	72		
Total		232 (13.4%)				25(1.4%)			

Considering the occurrence of *Campylobacter* infection in relation to growth characteristics, children that were stunted (13.7%) had higher *C. jejuni* infection than those that were not stunted (13.3%), but the difference was not statistically significant (χ^2^ = 0.061; *p* = 0.801). *C. coli* infection was more common in stunted (2.0%) children compared to children that were not stunted (1.2%), but the difference was also not statistically significant (χ^2^ = 1.610; *p* = 0.204) **(**Table 4).

### Sequence diversity of the hippuricase gene

**Fig. 4:**
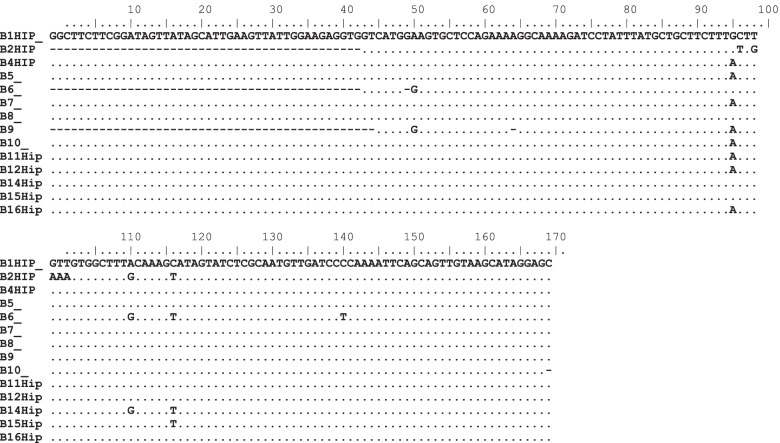
Nucleotide sequence of Hip gene (FJ655193 and KF54196) are referral strains from gene Bank

The nucleotide sequences of the hippuricase gene (14 *hip* isolates) were edited using Bioedit package, and aligned with two other sequences (FJ655193) and (KF541296) obtained from GenBank using the Clustal W multiple aligner. Sequence analysis confirmed sequences as belonging to the species *C. jejuni.* Figure 4 shows the nucleotide sequences of *Hip* genes with some mutations on various positions. Changes were found at different positions and appeared like single nucleotide polymorphisms these included positions 50, 95, 96, 98, 99, 100,101 110, 116, 140. Based on the alignment and comparison using referral strains (FJ655193 and KF54196) there were no two strains that were identical to each other. This depicts a wide sequence variation that could be existing in *Campylobacter jejuni* strains in this environment.

## Discussion

*Campylobacter* species are increasingly being recognised as agents of gastroenteritis worldwide with its prevalence varying from country to country [5, 6, 16, 17]. The current study aimed at determining the prevalence of *Campylobacter* infection in young children, and to ascertain the prevalence of *C. jejuni* and *C. coli* in the stool samples of cohort children in the Dzimuali community and the association of these pathogens with diarrheal and non-diarrheal samples and child growth.

*Campylobacter spp* were detected in 13.2% (564/4280) of the samples using ELISA. This is closely similar to reports generated from other countries. For example, in Algeria a prevalence of 17.7% was reported by Wren et al*.* [18], Nigeria (8.3%) [19], Tanzania (9.7%) [20], Bangladesh (19.3%) [21]. In Kenya, at a hospital in Kisii,5.8% of samples from patients with diarrheal were positive for *Campylobacter* species [22]. Also, in Egypt campylobacteriosis has been reported at (9%) [23]. These differences could be as a result of differences in geographical location, study population, study period and method employed for each study. Generally, the incidence of *Campylobacter species* (54.4%) infections was higher than that of *Campylobacter jejuni /coli*. This corroborates previous study which showed that promotion of, drinking treated water, improved latrines, and targeted antibiotic treatment may reduce the burden of *Campylobacter species* infection [16].

The higher prevalence of *Campylobacter* infection in children with diarrheal in this study may be due to unprotected water source and presence of domestic animals in almost all rural household. There was a high prevalence of *Campylobacter* in females than in male infants in this study. However, this is contrary to the reports of Coker et al*.* [1] who in their study among young children < 2yrs and young adults between the ages of 16-18yrs in Nigeria observed that *Campylobacter* infections are usually predominant in males. In Colorado, for example, males predominate in all age groups under 40 and the greatest difference between sexes was in infants where the male–female ratio was over 2:1 [1, 24].

Findings from this study showed higher *Campylobacter* infection in children between the age of five and eleven months (Fig. 1). Reports in developing countries show that isolation rates are higher among children less than one year of age with highest rates occurring in children between 6 to 12 months and do not increase in young adults [1, 25, 26]. The seasonal incidence of *Campylobacter* in this study is similar to that previously described by [27, 28] with a sharp rise in June and July. The period of June to July are early winter months in South Africa and the sharp rise could be in line with the explanation that milder winter temperatures may favour some transmission routes, as well as enhance the survival and multiplication of the bacteria [29]. However, is contrary with findings in most European countries where campylobacteriosis shows a marked seasonality peaks during the summer months [27] although it varies from country to country. Annually, consistent rise in incidence have been reported in the United Kingdom, Greece, the Netherlands, and Denmark [29]. A study in northwest England also indicated a consistent peak of human infection in March [28], which is the opposite of what has been observed in this study. The results obtained in this study showed that *Campylobacter* infection is endemic in the Vhembe district of South Africa since the prevalence remains almost at the same level throughout the 4 years.

In this study, more *C. jejuni* were detected than *C. coli* with a prevalence rate of 13.4% and 1.4%, respectively. Similar study in Venda region found that *C. jejuni* was higher (6.2%) than *C. coli* (3.1%) in children less than the age of five [8]. Again in Italy, Di Giannatale et al*. *[30], in their study, found that the prevalence of *C. jejuni* and *C. coli* was 62.75% and 37.25%, respectively. In a study on animals in Greater Washington, 53.6% of their isolates were *C. jejuni,* 41.3% were *C. coli* [31] which are in line with the findings of this study. Children that were stunted had higher *C. jejuni* and *C. coli* infection than those that were not. This corroborates previous reports which had it that, infection with specific enteric pathogens such as *Campylobacter spp.* can affect growth, even in the absence of overt diarrhea [16, 32, 33].

Several mutations were observed on the analysed *hip* gene (position 50, 95, 96, 98, 99, 100, 101, 110, 116, 140) which affected 12 participants out of 14. The mutation observed could be responsible for inability of some of the *Campylobacter* strains to induced clinical state of disease in some infants in this study. In this study, GGG was observed in children that showed no symptoms of *Campylobacter enteritis.* Phylogenetic analysis of the *hip* gene isolates from Venda region revealed that *Campylobacter* infection that had occurred among the children in this study were caused by non-related strains. From the distance matrix data, it is evident that *hip* genes that were sequenced show significant genetic differences.

## Conclusions

The present study indicates that infection caused by *Campylobacter* species was very frequent in children under the age of two in our study environment. *C. jejuni* was more prevalent in this study than *C. coli* and both species were significantly associated with diarrheal and might impact on growth decline in these children*.* These findings are of public health significance as it delineates the high frequency of *Campylobacter species* which might be associated with growth shortfall in children within the study area.

## Data Availability

Data generated or analysed during this study are included in this article. However, the absolute raw data are available from the corresponding author on request.
